# An N6-methyladenosine regulation- and mRNAsi-related prognostic index reveals the distinct immune microenvironment and immunotherapy responses in lower-grade glioma

**DOI:** 10.1186/s12859-023-05328-7

**Published:** 2023-06-01

**Authors:** Guihua Tang, Jianqiao Peng, Longwei Huo, Wen Yin

**Affiliations:** 1 Department of Clinical Laboratory, Hunan Provincial People’s Hospital (The first affiliated hospital of Hunan Normal University, The College of Clinical Medicine of Human Normal University), Changsha, 410005 Hunan Province People’s Republic of China; 2 Department of Neurosurgery, Yulin First Hospital Affiliated to Xi’an Jiao Tong University, Yulin, 719000 People’s Republic of China; 3 Department of Neurosurgery, National Clinical Research Center for Geriatric Disorders, Xiangya Hospital of Central South University, Changsha, 410008 Hunan Province People’s Republic of China

**Keywords:** Lower-grade glioma (LGG), N6-methyladenosine (m6A) regulation, mRNAsi, Prognostic index, Immune microenvironment, Immunotherapy responses, Nomogram

## Abstract

**Background:**

N6-methyladenosine (m6A) modification is involved in tumorigenesis and progression as well as closely correlated with stem cell differentiation and pluripotency. Moreover, tumor progression includes the acquisition of stemness characteristics and accumulating loss of differentiation phenotype. Therefore, we integrated m6A modification and stemness indicator mRNAsi to classify patients and predict prognosis for LGG.

**Methods:**

We performed consensus clustering, weighted gene co-expression network analysis, and least absolute shrinkage and selection operator Cox regression analysis to identify an m6A regulation- and mRNAsi-related prognostic index (MRMRPI). Based on this prognostic index, we also explored the differences in immune microenvironments between high- and low-risk populations. Next, immunotherapy responses were also predicted. Moreover, single-cell RNA sequencing data was further used to verify the expression of these genes in MRMRPI. At last, the tumor-promoting and tumor-associated macrophage polarization roles of TIMP1 in LGG were validated by in vitro experiments.

**Results:**

Ten genes (DGCR10, CYP2E1, CSMD3, HOXB3, CABP4, AVIL, PTCRA, TIMP1, CLEC18A, and SAMD9) were identified to construct the MRMRPI, which was able to successfully classify patients into high- and low-risk group. Significant differences in prognosis, immune microenvironment, and immunotherapy responses were found between distinct groups. A nomogram integrating the MRMRPI and other prognostic factors were also developed to accurately predict prognosis. Moreover, in vitro experiments illustrated that inhibition of TIMP1 could inhibit the proliferation, migration, and invasion of LGG cells and also inhibit the polarization of tumor-associated macrophages.

**Conclusion:**

These findings provide novel insights into understanding the interactions of m6A methylation regulation and tumor stemness on LGG development and contribute to guiding more precise immunotherapy strategies.

**Supplementary Information:**

The online version contains supplementary material available at 10.1186/s12859-023-05328-7.

## Introduction

Gliomas are heterogeneous neuroepithelial neoplasms deriving from the glial cells, which are the most common and lethal malignant brain tumor [1] in the central nervous system (CNS) [2]. Classically, they can be classified into grades I–IV based on their histopathological features. Diffuse grade II and III gliomas, including astrocytomas, oligodendrogliomas, and oligoastrocytomas, are considered lower-grade gliomas (LGG), and grade IV glioma is called glioblastoma multiforme (GBM) [3]. Due to the high infiltration and aggressiveness, LGGs are stubbornly resistant to first-line therapies referring to maximum neurosurgical removal followed by adjuvant radiotherapy and chemotherapy, ultimately, these tumors inevitably progress into a higher grade or experience relapse [4]. Nevertheless, because of the obvious intratumoral heterogeneity, the patients affected by LGG still have different biological and clinical characteristics, and their response to active therapy varies from person to person. Therefore, the median survival is showing an extreme range from 5.6 to 13.3 years [5]. Aimed at classifying patients more accurately, the genotypic features consisting of isocitrate dehydrogenase (IDH) mutation and 1p/19q co-deletion status were integrated into the traditional classification [5]. However, it provided valuable but insufficient and imprecise risk stratification and prognosis prediction, especially for genetically heterogeneous groups. Thus, it is urgent to uncover novel biomarkers to develop risk stratification and provide a new perspective for the personalized management of patients.

Recently, N6-methyladenosine (m6A) modification has gained increasing attention. It is the most prevalent and abundant form of modification in eukaryotic mRNA and is a dynamic reversible process regulated by a methyltransferase complex involved in binding proteins, methyltransferases, demethylases, also named “readers”, “writers”, and “erasers” [6]. Not only does m6A modification acts a vital role in mRNA metabolism ranging from RNA stability, splicing, export, intracellular distribution and translation [7], but also affects multiple biological processes such as regulating cell cycle and differentiation, and maintenance of circadian rhythm [8]. Additionally, the disorder of m6A regulators leads to weakened self-renewal capacity, developmental defects, dysregulation of cell proliferation, and cell death [7]. What is more, m6A modification is involved in many complex diseases, especially tumorigenesis and progression [9]. Alternatively, m6A methylation is also closely correlated with stem cell differentiation and pluripotency [10]. Excitedly, Tathiane M. Malta and colleagues [11] used machine learning to perform a multi-platform comprehensive analysis of 33 tumor types in The Cancer Genome Atlas (TCGA) database to obtain stemness indices that can quantify tumor stemness. It has also been demonstrated that higher mRNAsi values are accompanied by greater tumor dedifferentiation and more active biological processes related to cancer stem cells. Considering tumor progression includes the acquisition of stemness characteristics and accumulating loss of differentiation phenotype. Therefore, we integrated m6A methylation and stemness indicator mRNAsi to conduct a comprehensive analysis.

In the present study, first, we find genes related to m6A regulation and stemness index mRNAsi through consensus clustering and weighted gene co-expression network analysis (WGCNA). Next, the least absolute shrinkage and selection operator (LASSO) Cox regression analysis was used to identify an m6A regulation and mRNAsi-related prognostic index (MRMRPI), which could classify patients into different prognosis groups. Subsequently, we further explored the differences in immune status and immune microenvironment (such as immune cells, and immune pathways) between high- and low-risk populations. Additionally, the immunotherapy responses were predicted based on this MRMRPI. Then, a nomogram based on the MRMRPI was established to quantitatively predict prognosis. Finally, the biological function of one gene (TIMP1) in the MRMRPI was validated by in vitro experiments. It may be valuable to give novel insights into personalized management and fighting against LGGs by combining m6A and mRNAsi for providing promising prognostic targets.

## Results

### Identifying m6A methylation modification patterns of LGG

To explore whether the m6A methylation modification plays a vital role in LGG, consensus clustering was performed based on the expression of 21 regulators in TCGA and CGGA datasets. These regulators included 8 writers (CBLL1, KIAA1429, METTL3, METTL14, WTAP, ZC3H13, RBM15, RBM15B), 11 readers (ELAVL1, FMR1, HNRNPA2B1, HNRNPC, IGF2BP1, LRPPRC, YTHDF1, YTHDF2, YTHDF3, YTHDC1, YTHDC2), and 2 erasers (ALKBH5, FTO). It should be noted that IGF2BP1 is excluded from the CGGA analysis because of the lack of available sequencing data. In total, 481 samples in TCGA and 404 samples in CGGA with complete survival information were enrolled in the study. In TCGA cohort, patients were divided into three groups when *k* = 3 (Additional file 1: Fig. S1A-C), and there were significant differences in patient survival between different groups (Additional file 1: Fig. S1D, log-rank test). Meanwhile, similar results can be obtained in the CGGA cohort (Additional file 1: Fig. S1E-H).

### Identifying MRGs and screening modules related to mRNAsi by WGCNA

**Fig. 1:**
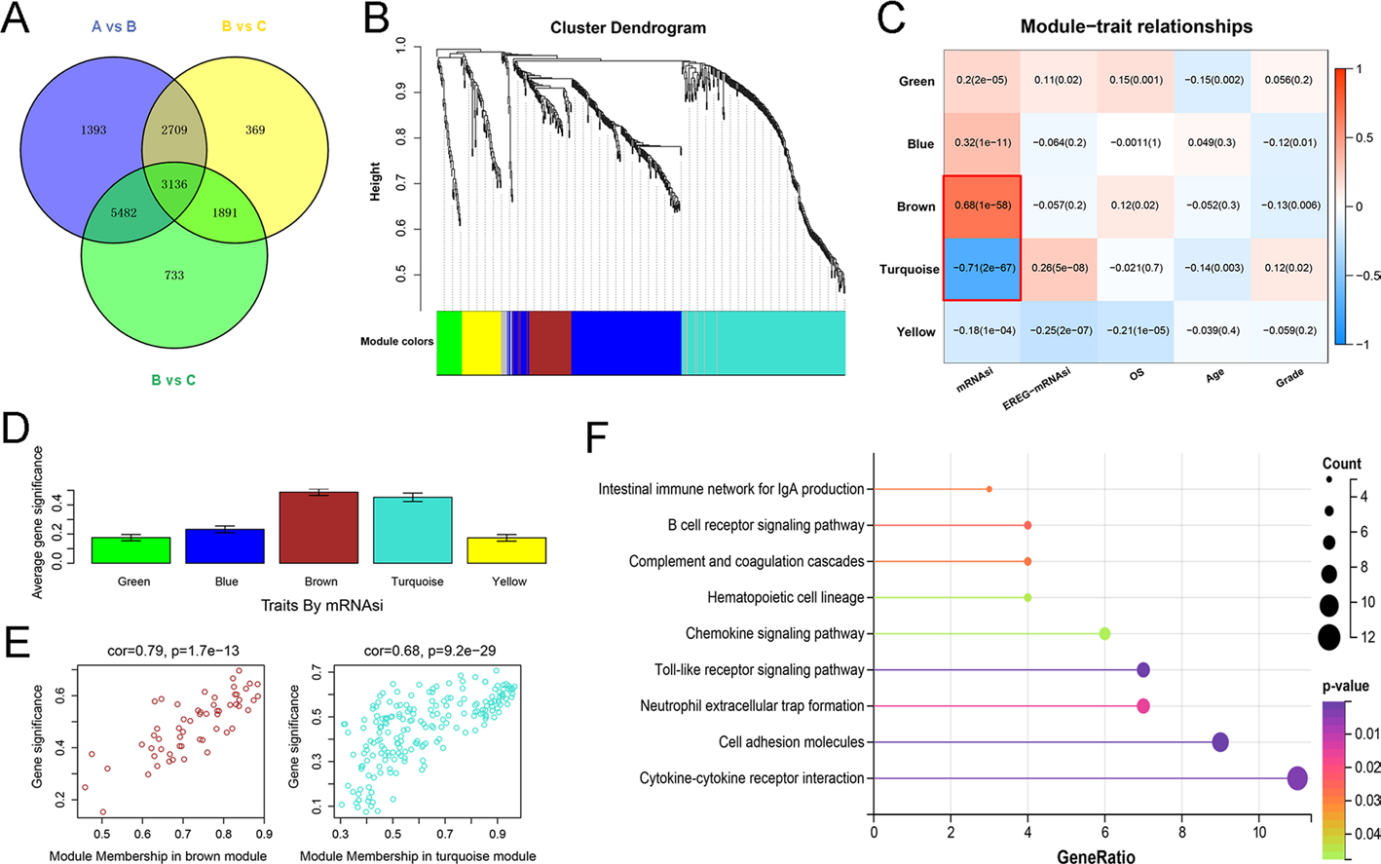
Screening of MRGs related to mRNAsi based on TCGA database. **A** The Venn diagram showed the common differential expressed MRGs between the three groups. **B** Weighted gene correlation network analysis (WGCNA) of the differential expressed MRGs. Different colors represent different modules. **C** Correlation analysis of the modules and clinical traits. Red-marked modules were selected for further analysis. **D** Gene significance of the mRNAsi trait.** E** Scatter plot analysis of modules in the brown and turquoise modules. **F** KEGG pathway analysis of the selected module genes

As shown in Fig. 1A, 3136 MRGs were identified. To further screen MRGs related to mRNAsi, WGCNA was performed to establish a co-expression network. We select β = 4 as the soft threshold to ensure a scale-free network after outlier samples are removed. Eventually, these MRGs were clustered into six modules, each with no less than 30 genes (Fig. 1B) and the genes non-clustered were assigned to the gray module. Next, the correlations between these modules and each phenotype were calculated separately as shown in Fig. 1C. Among these modules, the brown (positive correlation) and turquoise (negative correlation) modules attracted our attention due to the highest correlation with mRNAsi. At the same time, for mRNAsi, the gene significance of these two modules is the most remarkable (Fig. 1D). Besides, we plotted to scatter diagrams based on the GS and MM of each gene in the two modules (Fig. 1E), the high correlations between them revealed the importance of module genes and their close correlation with mRNAsi. Therefore, a total of 260 genes were selected, including 58 brown module genes and 202 turquoise module genes for subsequent analysis, and they were significantly enriched in immune-related signaling pathways (Fig. 1F).

### Development and validation of MRMRPI

To construct a robust MRMRPI, TCGA acted as the training set and CGGA as the validation set. First of all, the candidate genes in the training set were subjected to univariate Cox regression to determine the top 50 genes ranked by *P* value, which were further submitted to LASSO Cox regression analysis (Additional file 2: Fig. S2A). Finally, 10 prognostic genes were chosen for the risk-scoring system, and their univariate Cox results were visualized using a forest plot (Additional file 2: Fig. S2B). Consistently, the K–M curves with the median expression level of these genes as the thresholds also demonstrated their prognostic significance (Additional file 3: Fig. S3A). And they also showed the same prognostic trend in the CGGA data set (Additional file 3: Fig. S3B). The calculating formula of this risk-scoring system was equal to a line combination of the expression value of these genes and the optimal coefficients (Additional file 2: Fig. S2C) derived from LASSO regression. Based on this system, patients ranked by their risk score (RS) were distinguished into high- and low-risk groups according to the median RS value. Besides, the patients with high risk showed a significantly shortened survival time (Additional file 2: Fig. S2D). Simultaneously, aimed at validating the robustness of MRMRPI, the same RS formula and similar procedures were conducted in the validation set. 404 patients were classified into different groups, as expected, the one with low risk showed a significantly prolonged survival time (Additional file 2: Fig. S2E). Moreover, the ROC curves also revealed the good predictive performance of this MRMRPI in TCGA (Additional file 2: Fig. S2F) and CGGA (Additional file 2: Fig. S2G) cohorts. Finally, the DCA has also demonstrated the superiority of the MRMRPI in training (Additional file 2: Fig. S2H) and validation (Additional file 2: Fig. S2I) cohorts, which would make the clinical application more convincing.

### Subgroup analysis of immune landscape and relevant biological processes based on the MRMRPI

The result that screened module genes were significantly enriched in immune-related signaling pathways (Fig. 1F) such as cell adhesion molecules, Toll-like receptor signaling pathway, and so on, which indicated that the tumor immune microenvironment probably acted an important role in the LGG progress. Therefore, we further investigate the differences in the immune microenvironment between the high and low-risk groups of the MRMRPI. First of all, GSEA was performed in TCGA cohort, and results showed that there were 41 immune-related gene sets (Additional file 4: Fig. S4A) enriched in the high-risk subgroup but no one in the low-risk subgroup. The top 10 terms ranked by FDR were further displayed in Additional file 4: Fig. S4B.

**Fig. 2:**
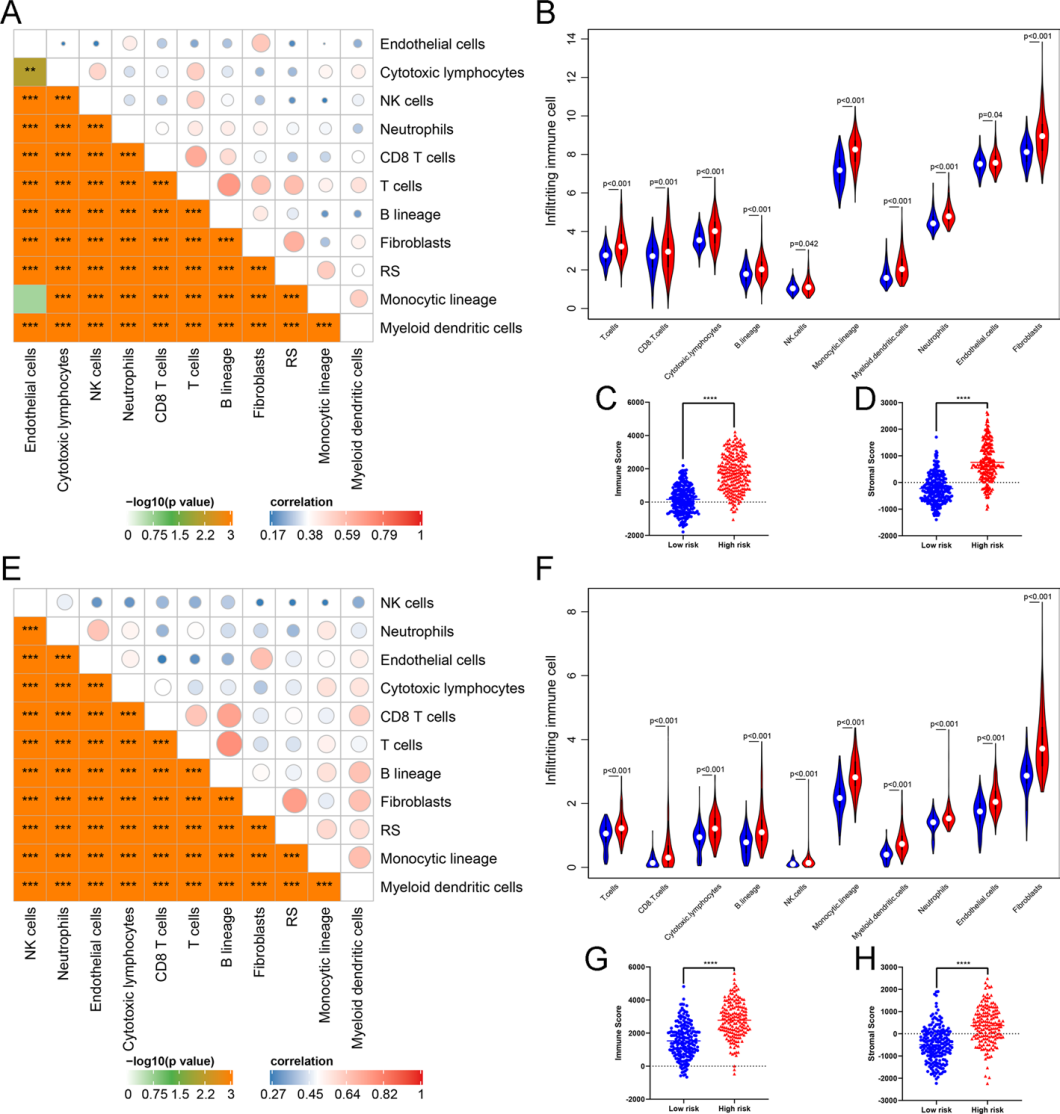
Immune infiltration in high-risk and low-risk groups. **A** Correlation matrix of risk score (RS and eight infiltrating immune and two stromal cell types in TCGA dataset. **B** Violin plot for comparing the fractions of eight infiltrating immune and two stromal cell types in high-risk and low-risk groups (TCGA dataset. **C** The comparison of immune scores in the high-risk and low-risk groups of TCGA dataset. **D** The comparison of stromal scores in the high-risk and low-risk groups of TCGA dataset. **E** Similar correlation matrix in the CGGA dataset. **F** Violin plot of infiltrating cells between high-risk and low-risk groups in the CGGA dataset. Immune **G** and stromal scores **H** in the high-risk and low-risk groups of the CGGA dataset

Afterward, we employed the ESTIMATE algorithm to calculate immune and stromal scores, and the MCP-counter algorithm to estimate the abundance of infiltrating immune cells (T cells, CD8^+^ T cells, cytotoxic lymphocytes, B lineage, NK cells, Monocytic lineage, Myeloid dendritic cells, Neutrophils), stromal cells (Endothelial cells, Fibroblasts). As the results showed (Fig. 2A, E), there was an obvious positive correlation between RS and infiltrating cells as well as various infiltrating cell subpopulations except for the statistically non-significant relationship between Endothelial cells and Monocytic lineage in TCGA set (Fig. 2A). Also, the abundance of each cell subpopulation was all significantly higher infiltrated in the high-risk group compared with the low-risk group (Fig. 2B, F). Consistently, the high-risk group showed significantly higher immune and stromal scores than the low-risk group (Student’s *t* test, *P* < 0.0001) in both TCGA and CGGA cohorts, which were respectively exhibited on the box plots of Fig. 2C, D, G, H. Simultaneously, we further explored the correlation between RS and the expression of several common immune checkpoints. Pearson’s correlation analysis indicated significantly positive correlations between RS and immune checkpoints including GZMB, CD27, CTLA-4, ICOS, LAG-3, OX40, PD-1, and PD-L2. The results originated from TCGA and CGGA cohorts were visualized in Additional file 5: Fig. S5A-H and S5I-P respectively.

**Fig. 3:**
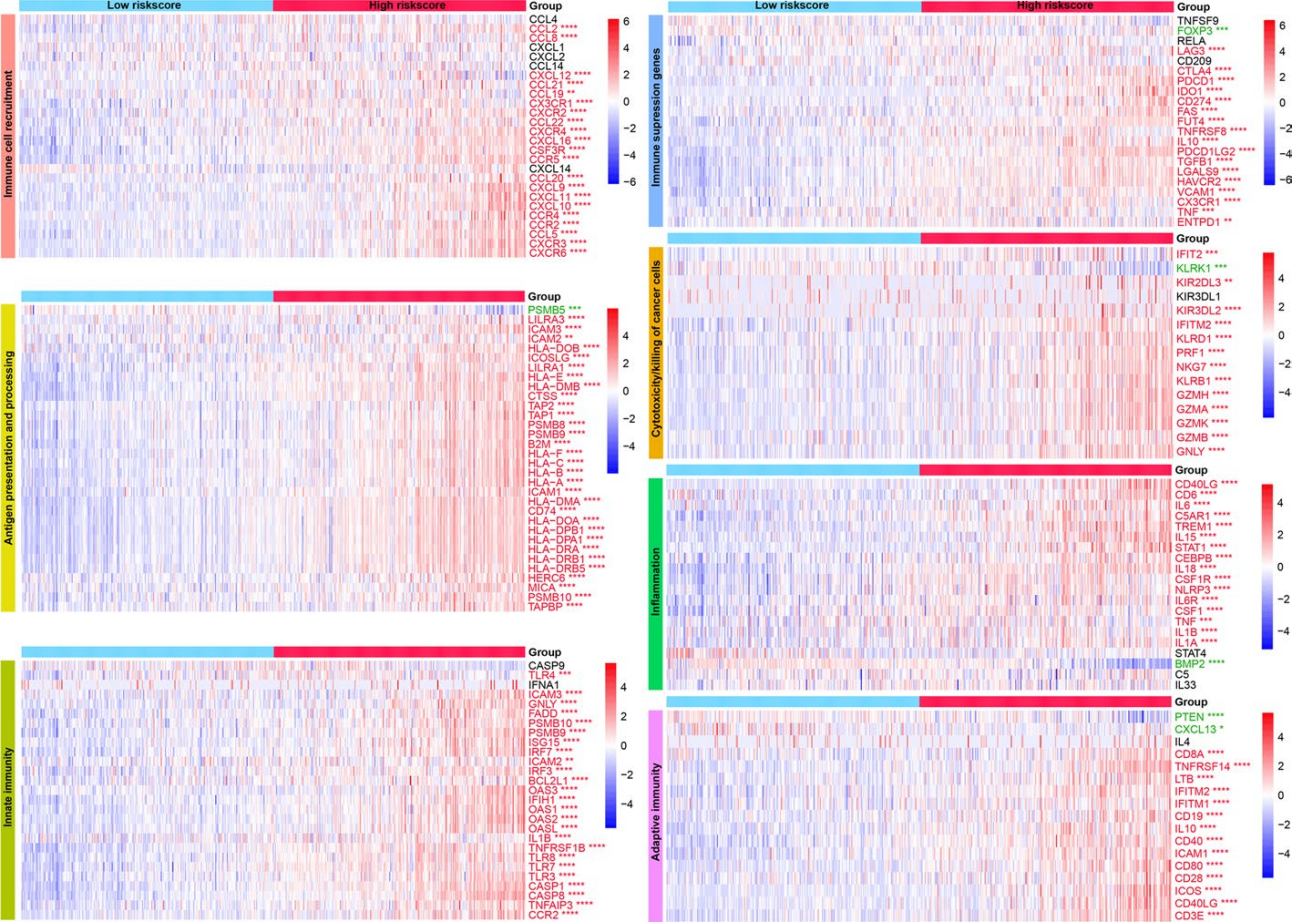
Activation of serveral immune pathways in the high-risk groups in the TCGA cohort. These pathways are involved in immune cell recruitment, antigen presentation and processing, innate immunity, immune suppression, cytotoxicity, inflammation, and adaptive immunity. The green font represents the gene overexpressed in the low-risk group, while the red represents the gene overexpressed in the high-risk group. Statistical test: Wilcoxon. *P < 0.05; **P < 0.01; ***P < 0.001; ****P < 0.0001

**Fig. 4:**
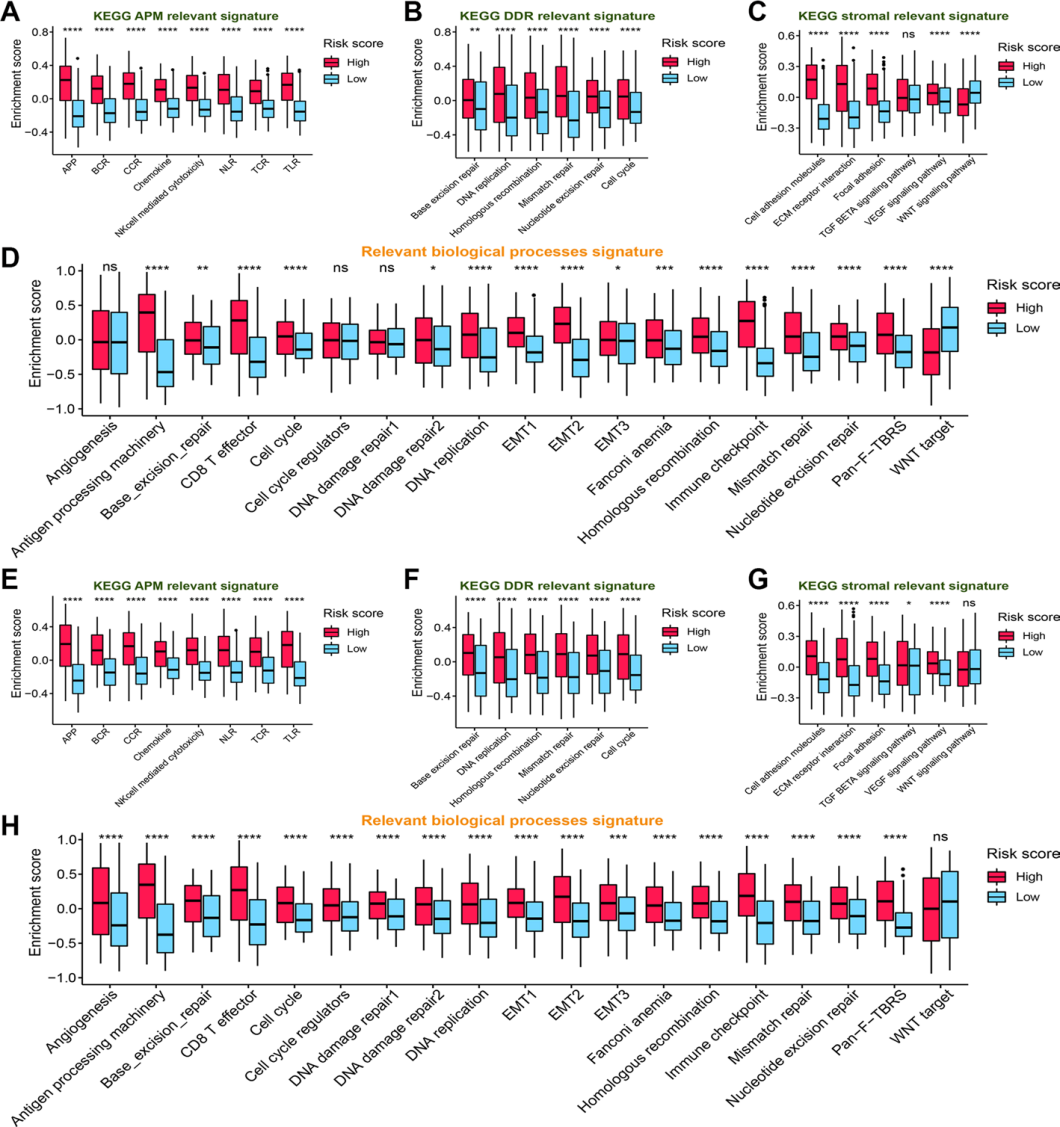
KEGG gene sets and biological process signatures in the distinct risk group. **A** KEGG APM relevant signature, **B** KEGG DDR relevant signature, **C** KEGG stromal relevant signature, and **D** relevant biological processes signature in high- and low-risk groups in the TCGA cohort. **E** KEGG APM relevant signature, **F** KEGG DDR relevant signature, **G** KEGG stromal relevant signature, and **H** relevant biological processes signature in high- and low-risk groups in the CGGA cohort. APM, antigen processing machinery; DDR, DNA damage repair

Next, to have a better understanding of the immune landscape and relevant biological processes between subgroups, we further annotated a series of gene signatures. The results showed that immune pathways related to immune cell recruitment, antigen processing and presentation, immune suppression, cytotoxicity, inflammation, and adaptive and innate immunity were obviously activated in the high-risk group both in TCGA (Fig. 3) and CGGA (Additional file 6: Fig. S6) cohorts. Consistently, functional annotation demonstrated that gene sets including immune activation, stromal activation, and DNA damage repair were remarkably enhanced in the high-risk group. This phenomenon was observed in both TCGA (Fig. 4A–D) and CGGA (Fig. 4E–H) cohorts.

### Chemotherapeutic drug sensitivity and immunotherapy response prediction

**Fig. 5:**
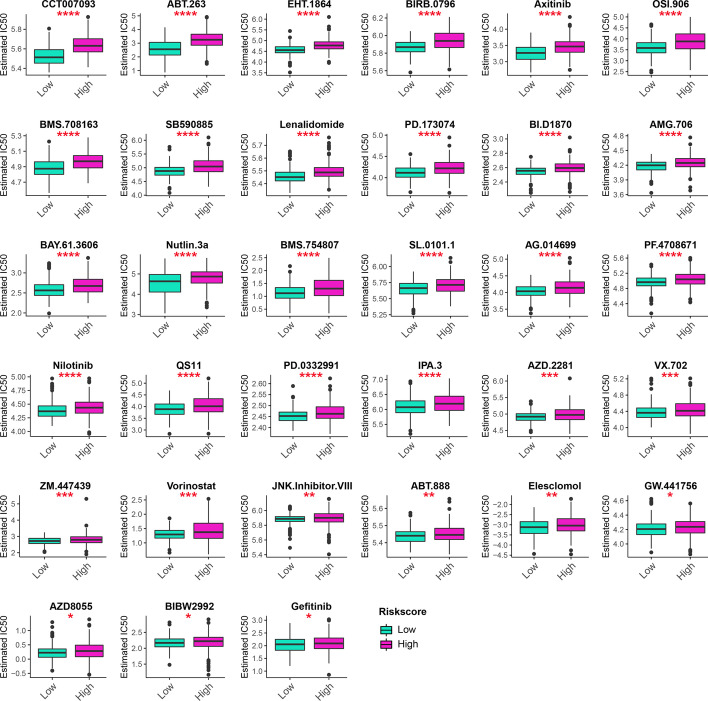
The 33 chemotherapeutic drugs, with significantly different IC50 between high- and low-risk groups, were identified by using "pRRophetic" package. Statistical test: Wilcoxon. *P < 0.05; **P < 0.01; ***P < 0.001; ****P < 0.0001

**Fig. 6:**
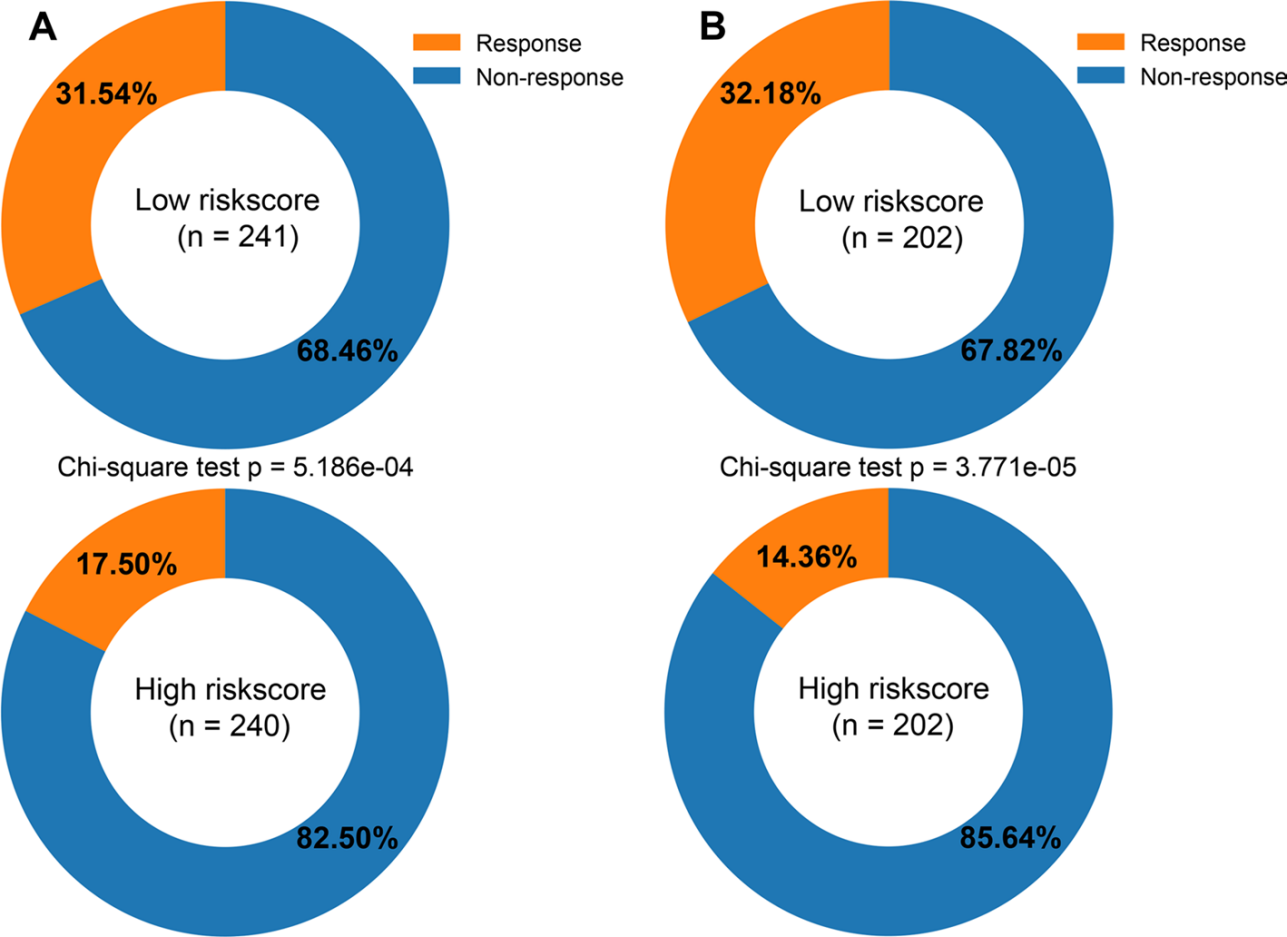
Prediction response to immunotherapy. Statistical test: Chi-square test. **A** Responders and non-responders for the low- and high-risk groups in the TCGA cohort. **B** Responders and non-responders for the low- and high-risk groups in the CGGA cohort

Chemotherapy is an important strategy for postoperative treatment; thus, we analyzed 138 chemotherapeutic drugs' sensitivity, and the results with statistically different IC50s between groups are shown in Fig. 5. Additionally, previous studies demonstrated that the tumor immune microenvironment plays important roles in tumorigenesis and immunotherapy [12, 13]. Considering that MRMRPI greatly affected the immune landscape and relevant biological processes, we inferred that the MRMRPI could be used to predict the responses to immunotherapy for LGGs patients. Eventually, the results that patients in the low-risk group responded significantly better than those in the high-risk group in both TCGA (Fig. 6A) and CGGA cohorts (Fig. 6B), which confirmed our speculation.

### Construction and assessment of the nomogram

**Fig. 7:**
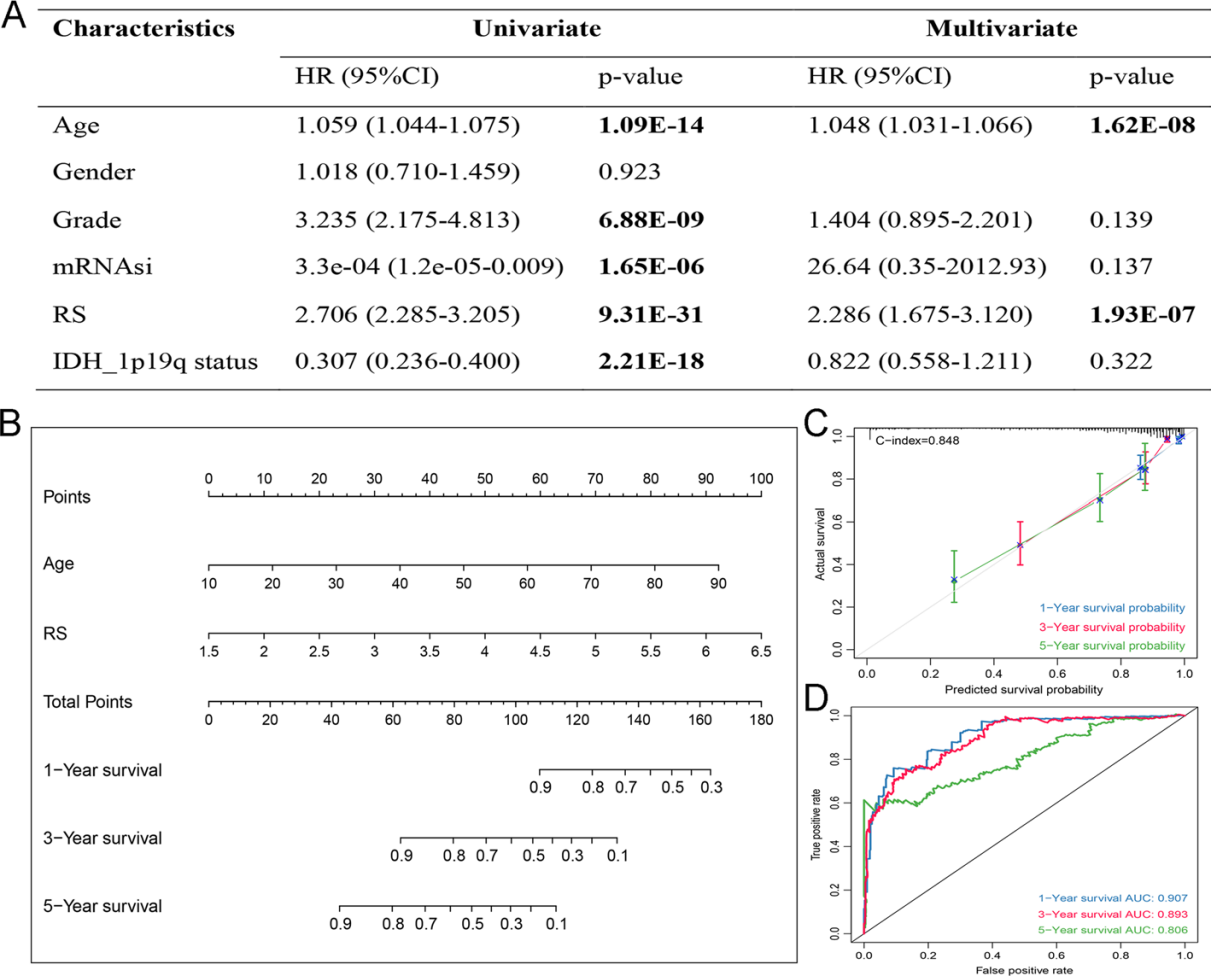
The nomogram for survival prediction in LGG patients based on TCGA dataset. **A** The results of univariate and multivariate Cox regression analysis. **B** The nomogram for the quantitative prediction of 1-, 3-, and 5-years overall survival of LGG patients. The 1-, 3-, and 5-year calibration curves **C** and time-dependent ROC curves **D** of the nomogram

To construct a nomogram that could quantitatively predict the survival probability of LGG patients, the independent prognostic risk factors were screened by univariate Cox analysis followed by multivariate Cox analysis in TCGA cohort. The RS and clinical characteristics including age, gender, grade, mRNAsi, and IDH_1p/19q status were incorporated into the above screening process. However, only the RS and age demonstrated independent prognostic values (Fig. 7A), which were finally integrated into the construction of the nomogram (Fig. 7B). The high C-index of 0.848 showed a good discrimination ability, and the calibrations presented strong coherence for the predicted and actual probabilities of 1-, 3-, and 5-year survival (Fig. 7C). Moreover, the ROC analyses also suggested an excellent predictive ability for sensitivity and specificity (Fig. 7D) with its 1-, 3-, and 5-year predicting AUC equal to 0.907, 0.893, and 0.806 respectively.

### Analysis of gene expressions at the single-cell level

**Fig. 8:**
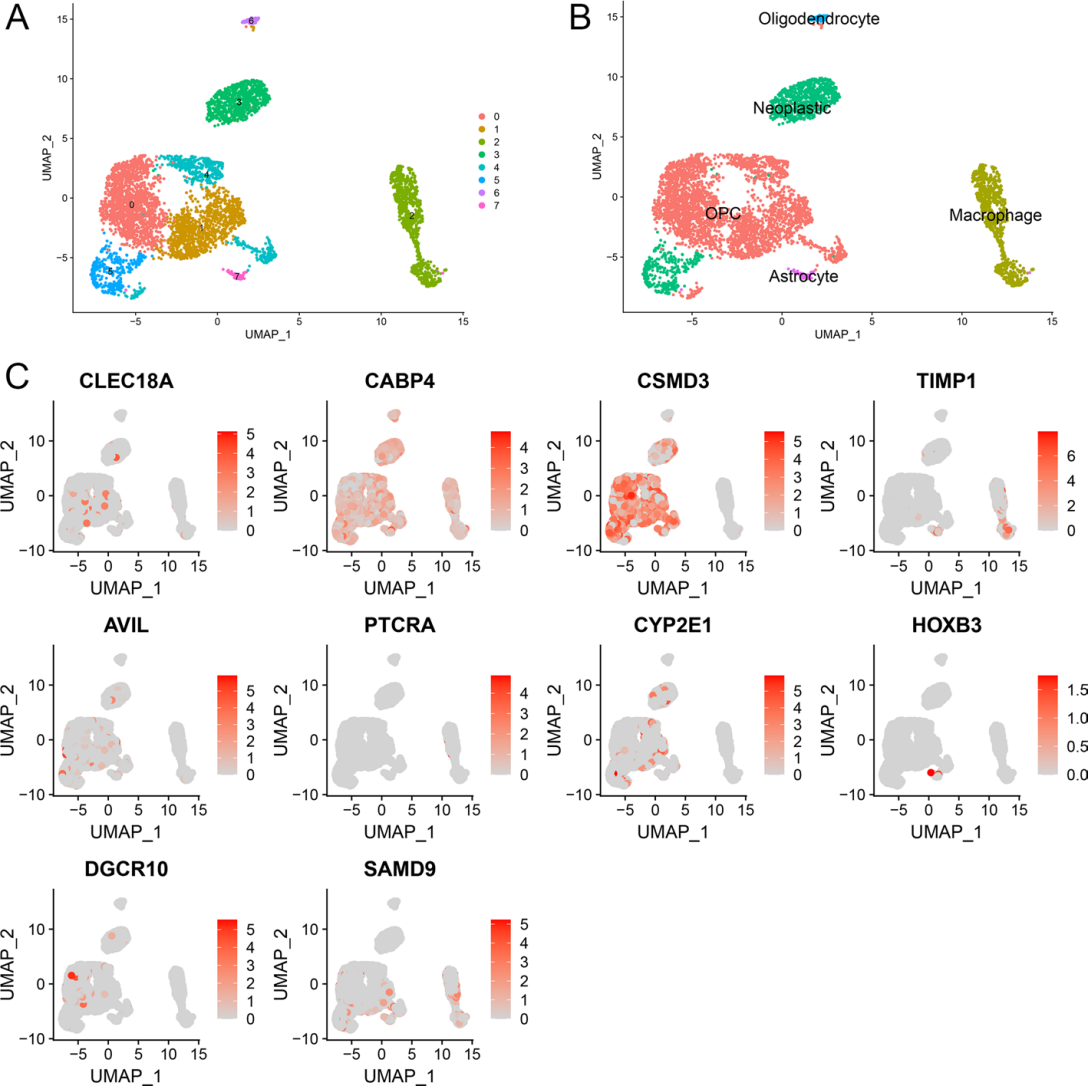
scRNA-seq results for the gene signature in LGGs. **A** The cells were classified into seven clusters based on UMAP. **B** Cell annotation by the “scCATCH” R package. **C** Visualizing the distribution of the gene signature in different cell types

To further analyze the expression of the gene signature at a single cell level, we performed scRNA-seq analysis in LGG. Totally, 7 clusters of cells were visualized by the UMAP dimensionality reduction algorithm (Fig. 8A). Moreover, 5 major cell types (neoplastic cells, oligodendrocyte precursor cells (OPCs), macrophages, astrocytes, and oligodendrocytes) were identified from the eight LGG samples (Fig. 8B). As Fig. 8C illustrated, genes like CSMD3, and CABP4 were expressed in most cell types, whereas TIMP1 was mostly expressed in macrophages and astrocytes. HOBX3 is almost only expressed in astrocytes. Gene like PTCRA was expressed very low in specific cell types.

### TIMP1 promotes the proliferation, migration and invasion of LGG cells and macrophage polarization

**Fig. 9:**
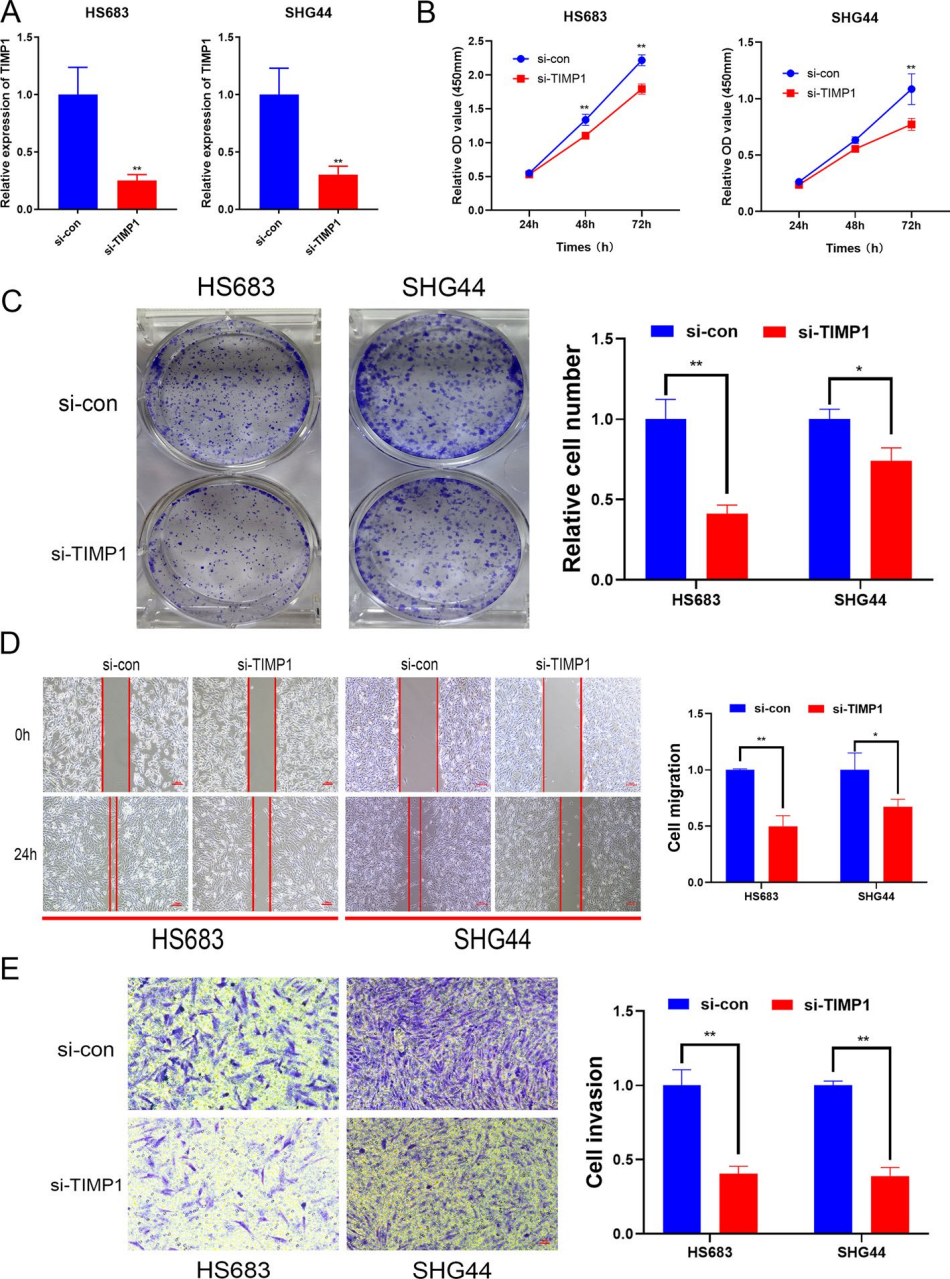
TIMP1 promotes the proliferation, migration and invasion of LGG cells. The siRNA of TIMP1 significantly inhibited the expression of TIMP1 in LGG cells **A**. The results of CCK-8 assays **B**, colony formation assays **C**, Wound healing assays **D**, and Transwell assay **E** revealed that the inhibition of TIMP1 expression remarkably suppressed the proliferation, migration, and invasion of LGG cells. *P < 0.05, **P < 0.01

Among the ten genes that make up the MRMRPI, the biological functions of TIMP1 in LGG remained unknown. To validate the tumor-promoting role of TIMP1 in the MRMRPI, siRNA was used to inhibited the expression of TIMP1 in HS683 and SHG44 cells (Fig. 9A). The CCK-8 assays and colony formation assays showed that the inhibition of TIMP1 significantly inhibited the proliferation ability of LGG cells (Figs. 9B, C). Meanwhile, the results of wound healing and Transwell assays demonstrated that knockdown of the expression of TIMP1 remarkably inhibited the migration and invasive capacity of LGG cells (Fig. 9D–E). These results illustrated that TIMP1 played a tumor-promoting role in LGG cells, and its risk role in the MRMRPI was validated.

In both HS683 and SHG44 cell lines, immunofluorescence staining analysis revealed that the fluorescence intensity ratio of CD163 and CD68 was considerably lower in the si-TIMP1 group than in the control group (Additional file 7: Fig. S7A). The statistical analyses were showed in Fig. S7B. These results showed that knocking down TIMP1 in glioma cells inhibited tumor-associated macrophage polarization.

## Discussion

Mounting evidence suggested that m6A modification serves a crucial role in cancer and non-cancerous diseases, aberrant m6A RNA methylation could affect inflammation, innate immunity as well as the response to antitumor therapy, and the interaction between regulatory factors can promote or suppress the progression of tumors [14]. As most studies concentrate on single or several regulators rather than comprehensive and overall analysis, to fill this insufficiency, we paid attention to MRGs based on distinct m6A modification patterns that demonstrated taking effect in tumors such as gastric cancer [15], colon cancer [16], hepatocellular carcinoma [17] as well as gliomas [18]. On the other hand, as well known that undifferentiated malignancies are responsible for tumor recurrence and anti-tumor resistance, and contribute to disease progression and poor prognosis. Recently, Malta et al. utilized a machine-learning algorithm [11] to develop a stemness index mRNAsi, which describes the similarity between stem and tumor cells according to gene expression data and possesses satisfactory adaptability and quantification of stemness. Besides, it was observed that mRNAsi was tightly associated with survival in several tumors including LGGs in pan-cancer cohorts. Hence, we combined MRGs and mRNAsi in the present study to develop a robust prognostic index for LGGs. As far as we know, it is the first study integrating MRGs and mRNAsi, which provides a novel insight into a more systematic understanding of LGG progression, facilitating more effective looking for therapeutic targets and enhancing more personalized management of LGGs.

In the present study, the m6A modification patterns classified by Consensus Clustering instead of just m6A regulators attracted our attention. Subsequently, MRGs were obtained from differential expressed analyses between various patterns, which could help us more comprehensively understand the role of m6A in LGG. Next, the WGCNA algorithm was used to integrate MRGs and mRNAsi, and two module genes were identified. Finally, there were ten genes incorporated into the development of the MRMRPI determined by univariate Cox regression and LASSO regression analyses. Among these genes, DGCR10, CYP2E1, and CSMD3 were identified as protective genes with their high expression closely associated with prolonged survival. In contrast, HOXB3, CABP4, AVIL, PTCRA, TIMP1, CLEC18A, and SAMD9 were confirmed as risk-associated genes. In previous publications, DGCR10 (also known as DGCR5) acted as a tumor-suppressive factor in glioma, consistent with our findings, which upregulated significantly inhibited glioma cell proliferation, migration, and invasion, whereas promoted apoptosis [19]. CYP2E1 genetic polymorphism affects the susceptibility of multiple tumors [20, 21], while CSMD3 mutation is significantly linked to prognosis in some cancers such as non-small cell lung cancer [22], esophageal squamous cell carcinoma [23], but their specific mechanism still needed further exploring. Regarding the risk-associated genes, previous studies demonstrated that HOXB3 promotes cell proliferation and invasion in glioblastoma [24], overexpressing a cytoskeleton regulator AVIL accelerate cell proliferation and migration, enables fibroblasts to transform into immortalized astrocytes to drive tumorigenesis of glioblastoma [25], but their roles in LGGs is still ambiguous. TIMP1 showed a positive correlation with glioma malignancy [26] and its expression level was suggested as an independent predictor of glioblastoma survival [27]. However, there was still a lack of experiments to validate the biological function of TIMP1. Herein, we performed in vitro experiment and the results showed that TIMP1 promotes the proliferation, migration and invasion of LGG cells. Moreover, the scRNA-seq analysis showed that TIMP1 is mostly expressed in macrophages, which maybe regulate the biological function of tumor-associated macrophages in the microenvironment of LGG. More importantly, in vitro experiments, we further confirmed the polarizing effect of TIMP1 on tumor-associated macrophages. Currently, there was almost no report about CABP4, PTCRA, CLEC18A, and SAMD9 in LGGs, which remains to be fully explored.

Then, we compared the immune status, as well as the distribution of immune scores, stromal scores, immune cells, immune pathways and relevant biological processes between high- and low-risk groups. Stromal scores in the high-risk group were higher than that in the low-risk group as expected, which was consistent with the reports that tumor stromal strongly facilitated the growth, progression, differentiation, and metastasis of tumor cells by nourishing tumor parenchyma [28]. Stromal-related signatures activated in the high-risk group also confirmed such a viewpoint. Interestingly, the immune scores, as well as infiltrated immune cells, were significantly higher in the high-risk rather than low-risk group. This may be related to the formation of an immune-excluded microenvironment. As reported that although such a microenvironment was abundant in immune cells, they stayed in the matrix surrounding tumor cell nests instead of penetrating parenchyma [15, 29]. The matrix may penetrate the tumor itself or be limited to the tumor envelope, which makes immune cells seem to be truly inside the tumor. Besides, the results that the immune checkpoints expressed much higher in the high-risk group are in accord with this assumption, which could contribute to the state of immunosuppression and lead to a poor prognosis [30]. Furthermore, antigen and inflammation persist within the tumor microenvironment, which will lead to T-cell exhaustion and dysfunction [12]. This explains the activation of inflammatory and immune pathways in the high-risk group rather than the low-risk group. In addition, the immune system needs to maintain immune homeostasis, which contributes to avoiding potential tissue damage and autoimmunity as well as generates a successful immune defense [31]. Our GSEA results presented significantly enriched immune-related GO terms in the high-risk group, reflecting an activated immune system in this population. However, the activated immune system of high-risk patients generated an adverse rather than beneficial effect on the prognosis of LGGs, which may be caused by the imbalance between suppressed and activated responses. This also revealed its microenvironment dominated by chronic inflammation and immunosuppression. Therefore, it might be a valuable immunotherapy strategy that maintains an equilibrium between amplifying and suppressing the immune response, which was supported by the results that better responses to immunotherapy of patients in the low-risk group.

Immunotherapy has ushered in a new era of cancer treatment, however, increasing reports [32, 33] suggest that cancer stem cells (CSCs) may potentially play an important role in treatment resistance and have been suggested to accelerate the progression and recurrence of tumors. It has been shown that in gliomas, CSCs evade immune clearance by activating regulatory T (Treg) cells, inactivating dendritic and natural killer cells [34], suppressing T cell proliferation and recruiting infiltration of type 2 macrophages (M2) [35], which results in local or systemic immunosuppression [36]. Alternatively, tumor-associated macrophages (TAMs) secrete cytokines to enhance the self-renewal [37] of CSCs as well as to stimulate invasion and drug resistance [38] of CSCs. Therefore, strategies developed to target the stemness of tumor cells may identify new therapeutic opportunities for glioma treatment. We also established a quantitative prognostic prediction nomogram that combined the MRMRPI and independent clinical prognostic factors determined by univariate and multivariate Cox analyses. According to this nomogram, clinicians can classify patients into distinct risk stratification more accurately and provide more scientifically personalized management of LGG patients.

## Conclusion

In conclusion, as far as we know, it was the first time to construct an MRMRPI in LGG that integrated MRGs and mRNAsi, classifying patients into distinct risk stratification. Importantly, patients in different risk stratifications have completely different prognoses and immune microenvironment. These findings provide novel insights into the understanding interactions of m6A methylation regulation and tumor stemness on LGG development and contribute to guiding more precise immunotherapy strategies.

## Materials and methods

### Data collection and processing

The LGGs mRNA-Seq data was extracted from TCGA and Chinese Glioma Genome Atlas (CGGA) databases. The normalized former by log_2_(x + 1) transformed, along with relevant clinical information were downloaded from the UCSC Xena website (https://xena.ucsc.edu/), while the latter and corresponding clinical data were downloaded from the CGGA official website (http://www.cgga.org.cn/index.jsp). Only samples with complete survival data and survival time greater than 30 days were selected for subsequent analysis. At the same time, the sequencing data in CGGA is also log2(x + 1) transformed. Besides, the stemness index mRNAsi corresponding to the sample in TCGA-LGG was obtained from the previous research [11].

### Consensus clustering and differential expression analysis

To investigate whether the m6A regulation patterns are suitable for LGG, we mined the 21 regulators involving 8 writers, 11 readers, and 2 erasers if possible from a current study [15] to identify subtypes by Consensus Clustering analysis [39] in TCGA and CGGA datasets. 1000 times repetitions and Pearson correlation were conducted using the “ConsensuClusterPlus” package.

To screen m6A-related genes (MRGs), the “limma” R package was utilized in TCGA dataset to determine differential expression genes (DEGs) between different subtypes classified by the consensus clustering of m6A regulators expression [15]. Adjusted *P* value < 0.01 was defined as the significant criteria for identifying DEGs, and the overlaps are considered to be MRGs.

### Weighted gene co-expression network analysis (WGCNA)

The overlap of DEGs was used to establish a co-expression network [40, 41] in TCGA cohort and identify the modules most related to mRNAsi. Primarily, a gene expression similarity matrix was constructed based on Pearson correlation. Next, a proper soft-thresholding power β = 4 was selected to establish a signed weighted adjacency matrix, which was subsequently translated into a topological overlap matrix (TOM). Finally, the average linkage hierarchy was clustered with the parameter height = 0.25 and gene modules were identified. Besides, the module and clinical traits correlations were calculated by module eigengene (ME) representing the first principal component of each module. Next, gene significance (GS) the correlation between genes and clinical traits as well as module membership (MM) the correlation between module genes and gene expression profiles, which were both calculated. The high correlation between GS and MM means the importance of the genes in the module and the close correlation with the clinical trait.

### KEGG pathways

To explore the underlying biological functions of the chosen module genes, the Kyoto encyclopedia of genes and genomes (KEGG) enrichment analysis [42–46] was completed by using the “clusterProfiler” package. The significant cut-off value was defined as *P* < 0.05.

### Development and validation of the MRMRPI

After WGCNA filtering, the module genes related to m6A and mRNAsi were subjected to further analysis. Aimed at constructing an MRMRPI, of which the development was in TCGA set, while validation in CGGA set. First, univariate Cox regression was employed in the training set to screen the top 50 genes ranked by *P* value. Then, to identify the optimal genes with prognostic value and corresponding coefficients, the least absolute shrinkage and selection operator (LASSO) Cox analysis was performed just like the present studies [3, 47, 48]. Moreover, the MRMRPI was validated in CGGA dataset by using the same coefficients. Patients were classified into high- and low-risk subgroups based on the median cutoff value, which was proven by Kaplan–Meier (K–M) analysis with the log-rank test. Besides, time-dependent receiver operating characteristic (ROC) curves and decision curve analysis (DCA) were applied to evaluate the prognosis predicting performance.

### Functional and pathway enrichment analyses

To investigate the immune status variations between different risk groups, we performed GSEA with program gsea-3.0.jar, and the immune-related gene ontology gene sets were regarded as the reference gene sets, which were obtained from the Molecular Signatures Database (http://software.broadinstitute.org/gsea/msigdb/). The enrichment items are consideredsignificant only when the nominal *P* value < 0.01 and the false discovery rate (FDR < 0.25). We also performed a GSVA (gene set variation analysis) algorithm to identify the differences in biological processes between distinct groups. The gene sets included c2.cp.kegg.v7.4.symbols downloaded from the Molecular Signatures Database and other relevant biological processes retrieved from the supplementary material in a previous study [49].

### ESTIMATE algorithm and MCP-counter

ESTIMATE algorithm [50] was used to calculate the immune score and stromal score that could represent all immune cells and stromal cells respectively [51]. The Microenvironment Cell Populations-counter (MCP-counter) method was applied to further estimate the infiltrating cell types. It is a validated method that enables the reliable quantification of the abundance of 8 immune and 2 matrix populations in the transcriptome of malignant tissues, and the abundance of these cells allows an inter-sample comparison [52].

### Prediction of chemotherapeutic drug sensitivity and immunotherapy responses

To assess the predictive capability of MRMRPI in chemotherapeutic agent sensitivity, we calculated the half-maximal inhibitory concentrations (IC50) of 138 chemotherapeutic components by uing the "pRRophetic" package [53, 54] and compared the differences between the low- and high-risk groups. To further predict the response of immune checkpoint blockade (ICB) therapy, the ImmuCellAI platform (http://bioinfo.life.hust.edu.cn/ImmuCellAI/#!/analysis), a powerful tool [55] with high accuracy, was employed.

### Nomogram construction and validation

To strengthen the predictive performance of MRMRPI and quantitatively predict prognosis, we developed a nomogram [56] in TCGA dataset, which integrates RS derived from MRMRPI and other important prognostic biomarkers. Univariate and multivariate Cox regression analyses were employed to determine independent prognostic biomarkers incorporated into the nomogram. Additionally, the concordance index(C-index), calibration curves, and ROC curves were used to evaluate the nomogram.

### Single-cell RNA sequencing

According to a previous study, single-cell RNA sequencing (scRNA-seq) analysis was performed [57]. We downloaded a glioma dataset (GSE89567) in the GEO database and extracted the single-cell data expression matrix of LGG. The R package Seurat was employed to analyze the single-cell data. First, a Seurat object to store the data matrix was created by the“CreateSeuratObject” function. Then, quality control was performed to discard features and cells that do not meet the basic standards: (1) genes detected in more than 3 cells; (2) cells with more than 200 total detected genes; (3) cells with less than 5% of mitochondrial genes. Next, the “NormalizeData” function was used to normalize the data, and the function“FindVariableGenes” was applied to identify 2000 highly variable genes. Then, the principal component analysis (PCA) was performed. Afterward, a uniform manifold approximation and projection (UMAP) algorithm was used for further visualization. Finally, the “scCATCH” R package combined with manual annotation was used to annotate the cell types and “FeaturePlot” was used to visualize expressions.

### Cell culture and quantitative real-time PCR

LGG cell lines (HS683 and SHG44) were obtained from Xiangya School of Medicine, Central South University, Changsha, China. HS683 and SHG44 cells were cultured in high-glucose DEME (Gibco) supplemented with 10% fetal bovine serum. The Small interfering RNAs (siRNAs) against the TIMP1 gene were synthesized by RiboBio Corporation (Guangzhou, China). We used Lipofectamine 2000 transfection reagent (Invitrogen) for the siRNA transfection according to the manufacturer's protocol. The siRNA of TIMP1 (sense: CCACCUUAUACCAGCGUUATT, antisense: UAACGCUGGUAUAAGGUGGTT). The TRIzol lysis method was utilized to extract total RNA from cells. The Thermo Scientific RevertAid First Strand cDNA Synthesis Kit was used to synthesize cDNAs. The mRNA level of TIMP1 was detected by quantitative real-time PCR (qRT-PCR). The 2-ΔΔCt method was used to calculate the mRNA expression levels. The qRT-PCR primers were synthesized by Sangon Biotech (Shanghai, China), and the sequences were as follows: for TIMP1, the forward primer was 5ʹ-CTTCTGCAATTCCGACCTCGT-3ʹ and the reverse primer was 5ʹ-ACGCTGGTATAAGGTGGTCTG-3ʹ for GAPDH, the forward primer was 5ʹ-CATTGACCTCAACTACATGGTT-3ʹ and the reverse primer was 5ʹ-CCATTGATGACAAGCTTCCC-3ʹ.

### Wound healing and Transwell assays

The wound healing and Transwell assays were performed by previously described methods [58].

### Cell colony formation assay

After transfected with TIMP1 or control siRNAs, about 1000 cells/well were plated into 6-well plates and cultured for two weeks to allow colony formation. The colonies were fixed with 4% paraformaldehyde and stained with 0.01% crystal violet. Then we judged the cell growth ability according to the colony numbers.

### Cell proliferation assay

After HS683 and SHG44 cells were transfected with TIMP1 or control siRNAs, the Cell Counting Kit-8 (CCK-8) assay (Vazyme, Nanjing, China) was conducted to monitor cell proliferation ability. HS683 and SHG44 cells (1.5 × 10^3^ cells/well) were seeded into 96-well plates. Then, 10 μL of CCK-8 reagent was added to each well and incubated for 2 h at 37 °C. The Optical Density (450 nm) was determined on 0, 24, 48, and 72 h.

### Macrophage differentiation and co-culture system

Macrophages (M0) were induced by THP-1 monocytes. Briefly, the THP-1 cells were seeded into the 6-well plate at 1 × 10^6^ cells/ml. Then, 100 ng/ml PMA (Phorbol-12-myristate-13 acetate) was added for 48 h to obtain macrophages (M0). To establish a co-culture system, HS683 and SHG44 cells were seeded on top of the culture inserts, and macrophages (M0) were seeded in a 24-well plate. Subsequently, the macrophages (M0) were harvested for further analysis after 72 h.

### Immunofluorescence staining

Immunofluorescence staining was conducted to observe the expression level of CD68 and CD163. After discarding the culture medium, macrophages growing in a 24-well plate were fixed with 4% paraformaldehyde at room temperature for 10 min and then permeabilized by 0.3% Triton X-100 for 20 min. Next, at room temperature, 5% BSA (Bovine Serum Albumin) was used to block the unspecific binding sites for 2 h. The cells were incubated overnight with the primary CD68 (1:100; mouse; Proteintech 66,231–2-Ig) and CD163 (1:100; rabbit; Proteintech 16,646–1-AP) antibodies. Then, at room temperature, the slides were incubated in Alexa Fluor 568-conjugated donkey anti-mouse secondary antibody (1:500, Invitrogen) and Alexa Fluor 488-conjugated donkey anti-rabbit secondary antibody (1:500, Invitrogen) for 1 h. DAPI (1:500, Sigma, United States) was used to label the nuclei.

### Statistical analysis

Statistical analyses were carried out using the R software (version 4.0.0) and GraphPad Prism (version 8.0). The log-rank test was employed in the Kaplan–Meier survival analysis. Pearson’s correlation analyses were conducted to calculate the correlation between the two groups. Student's *t* test was used in the two-group comparisons. A *P* value less than 0.05 was considered statistically significant.

## Supplementary Information

**Additional file 1: Figure S1.** The consensus clustering of m6A regulators could classify LGG patients into three groups in TCGA and CGGA glioma datasets. (A) Consensus clustering matrix of 481 samples from TCGA dataset for *k* = 3. (B) Relative change in area under the cumulative distribution function (CDF) curves according to different k values (TCGA). (C) Principal component analysis (PCA) based on the expression of m6A regulators showed distinct groups of glioma patients (TCGA). (D) Survival analysis of patients in different groups in TCGA cohort. (E) Consensus clustering matrix of 404 samples from the CGGA dataset for *k* = 3. (F) Relative change in area under the CDF curve according to different k values (CGGA). (G) PCA based on the expression of m6A regulators showed distinct groups of glioma patients (CGGA). (H) Survival analysis of patients in different groups in the CGGA cohort.

**Additional file 2: Figure S2.** Construction and validation of the m6A regulation and mRNAsi-related prognostic index (MRMRPI). (A) The 10 genes were selected by least absolute shrinkage and selection operator (LASSO) Cox analysis in TCGA dataset. (B) Forest plot of the univariate Cox results of the 10 genes. (C) Coefficient values for each gene in the LASSO Cox analysis. Risk scores, living status, and Kaplan-Meier curves in the training (D) and validation cohorts (E). Time-dependent ROC curve analysis of the MRMRPI in the training (F) and validation (G) cohorts (H).

**Additional file 3: Figure S3.** Kaplan-Meier curves of the 10 prognostic genes in TCGA (A) and CGGA (B) datasets.

**Additional file 4: Figure S4.** GSEA showed the immune-related GO terms between low- and high-risk groups. (A) A total of 41 immune-related GO terms were significantly enriched in the high-risk group. (B) The visualization of the top 10 enrichments in the high-risk group.

**Additional file 5: Figure S5.** The correlation analyses between risk score (RS) and immune checkpoints in TCGA (A-H) and CGGA dataset (I-P).

**Additional file 6: Figure S6.** Activation of several immune pathways in the high-risk groups in the CGGA cohort. These pathways are involved in immune cell recruitment, antigen presentation and processing, innate immunity, immune suppression, cytotoxicity, inflammation, and adaptive immunity. Green font represents the gene overexpressed in the low-risk group, while red represents the gene overexpressed in the high-risk group. Statistical test: Wilcoxon. *, *p* < 0.05; **, *p* < 0.01; ***, *p* < 0.001; ****, *p* < 0.0001.

**Additional file 7: Figure S7.** TIMP1 promotes macrophage differentiation toward M2 in vitro. (A) The expression of CD68 and CD163 in macrophages treated differently detected by immunofluorescence. (B) Statistical analysis of different groups (** Represents *p* < 0.01).

## Data Availability

The datasets included in this study can be downloaded from public repositories including the UCSC Xena website (https://xena.ucsc.edu/) and the CGGA official website (http://www.cgga.org.cn/index.jsp).

## Abbreviations

CNS: Central nervous system
LGG: Lower-grade gliomas
IDH: Isocitrate dehydrogenase
m6A: N6-methyladenosine
TCGA: The Cancer Genome Atlas
WGCNA: Weighted gene co-expression network analysis
LASSO: Least absolute shrinkage and selection operator
MRMRPI: M6A regulation and mRNAsi related prognostic index
CGGA: Chinese Glioma Genome Atlas
MRGs: M6A-related genes
DEGs: Differential expression genes
TOM: Topological overlap matrix
ME: Module eigengene
GS: Gene significance
MM: Module membership
KEGG: Kyoto encyclopedia of genes and genomes
K–M: Kaplan–Meier
ROC: Receiver operating characteristic
GSEA: Gene set enrichment analysis
FDR: False discovery rate
MCP-counter: Microenvironment cell populations-counter
C-index: Concordance index
RS: Risk score
