# Supplementary figures and images for: An N6-methyladenosine regulation- and mRNAsi-related prognostic index reveals the distinct immune microenvironment and immunotherapy responses in lower-grade glioma
Source: BMC Bioinformatics. 2023 Jun 1;24:225. doi: 10.1186/s12859-023-05328-7 (PMC10236841; doi:10.1186/s12859-023-05328-7)

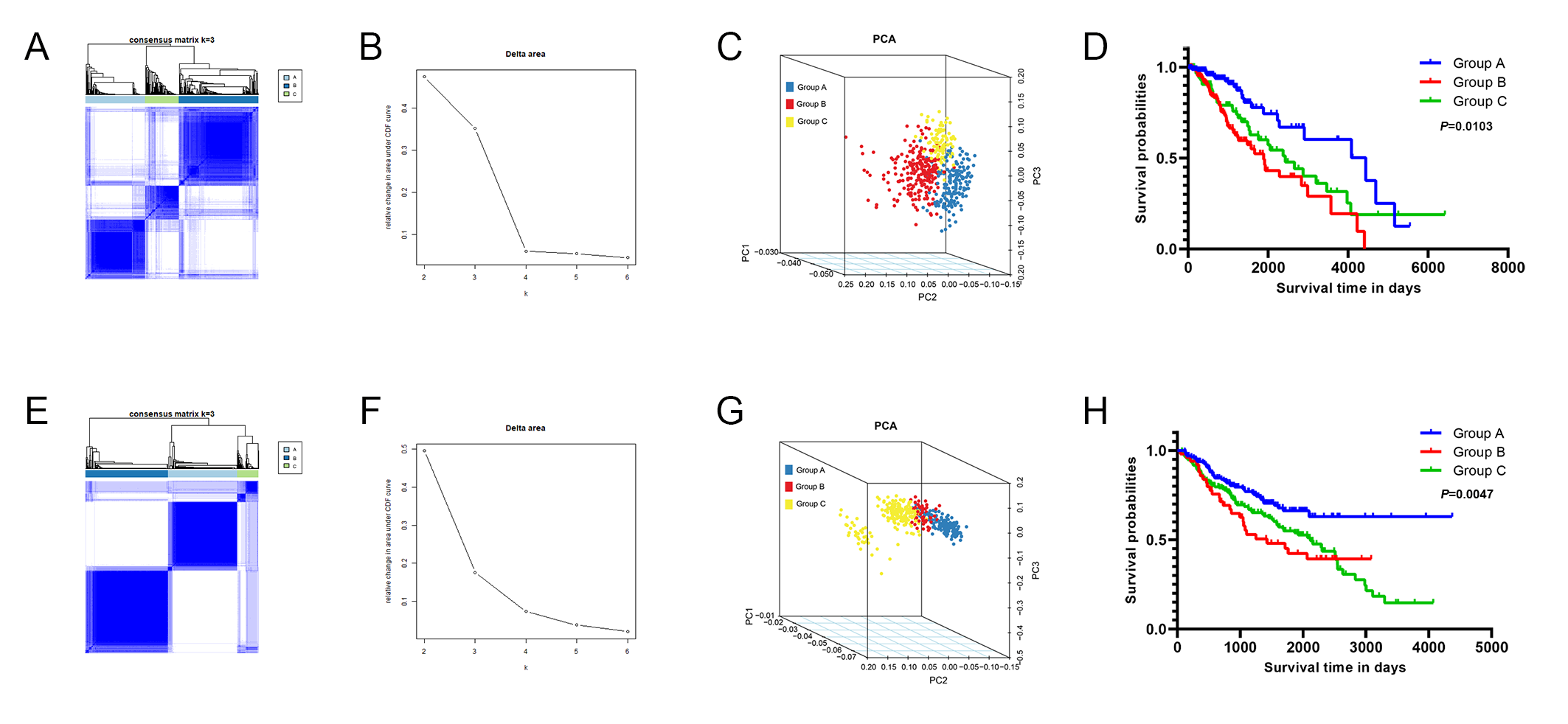

Supplement: Supplementary file 1 — Additional file 1: Figure S1. The consensus clustering of m6A regulators could classify LGG patients into three groups in TCGA and CGGA glioma datasets. (A) Consensus clustering matrix of 481 samples from TCGA dataset for k = 3. (B) Relative change in area under the cumulative distribution function (CDF) curves according to different k values (TCGA). (C) Principal component analysis (PCA) based on the expression of m6A regulators showed distinct groups of glioma patients (TCGA). (D) Survival analysis of patients in different groups in TCGA cohort. (E) Consensus clustering matrix of 404 samples from the CGGA dataset for k = 3. (F) Relative change in area under the CDF curve according to different k values (CGGA). (G) PCA based on the expression of m6A regulators showed distinct groups of glioma patients (CGGA). (H) Survival analysis of patients in different groups in the CGGA cohort. [file 12859_2023_5328_MOESM1_ESM.tif]

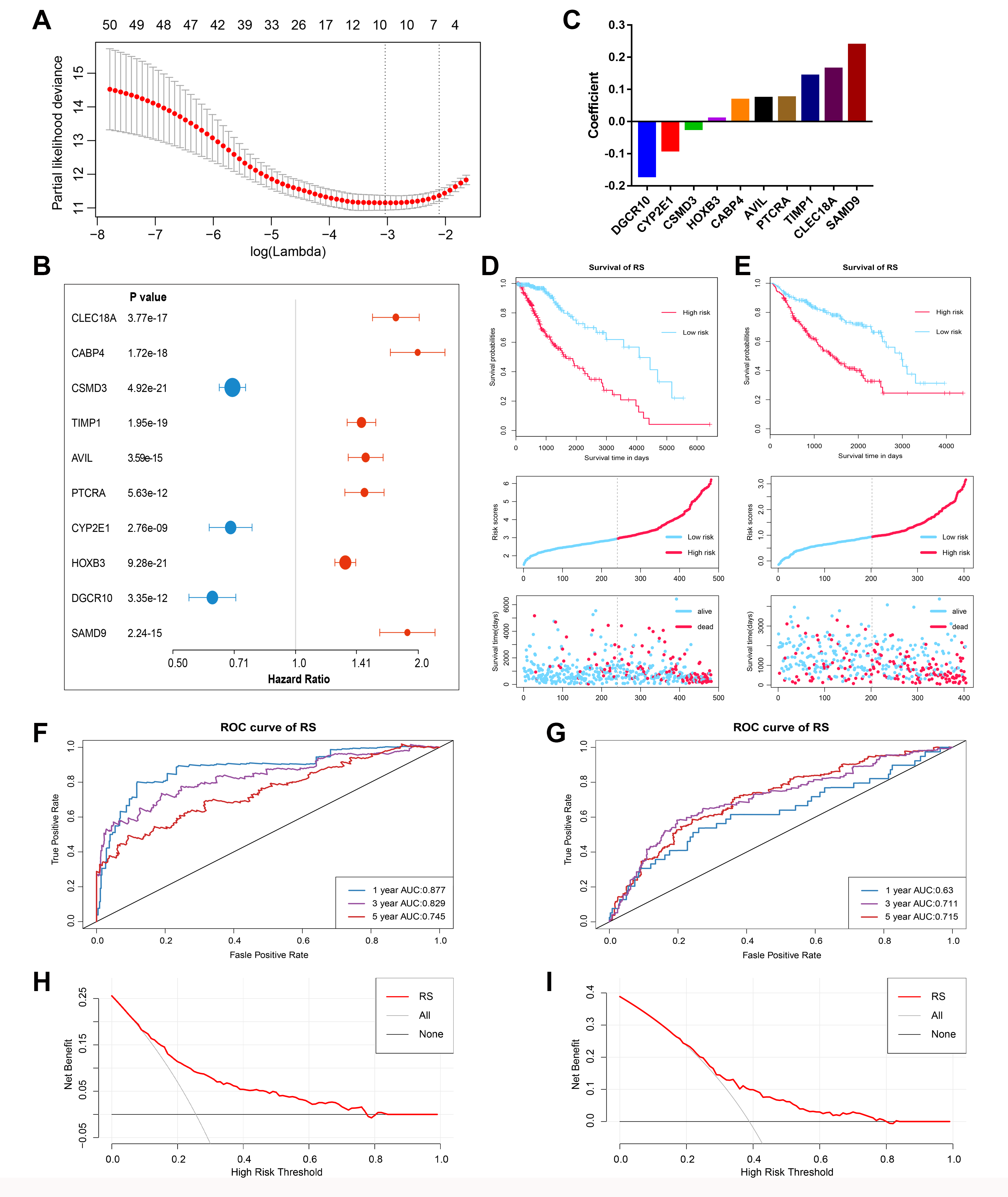

Supplement: Supplementary file 2 — Additional file 2: Figure S2. Construction and validation of the m6A regulation and mRNAsi-related prognostic index (MRMRPI). (A) The 10 genes were selected by least absolute shrinkage and selection operator (LASSO) Cox analysis in TCGA dataset. (B) Forest plot of the univariate Cox results of the 10 genes. (C) Coefficient values for each gene in the LASSO Cox analysis. Risk scores, living status, and Kaplan-Meier curves in the training (D) and validation cohorts (E). Time-dependent ROC curve analysis of the MRMRPI in the training (F) and validation (G) cohorts (H). [file 12859_2023_5328_MOESM2_ESM.tif]

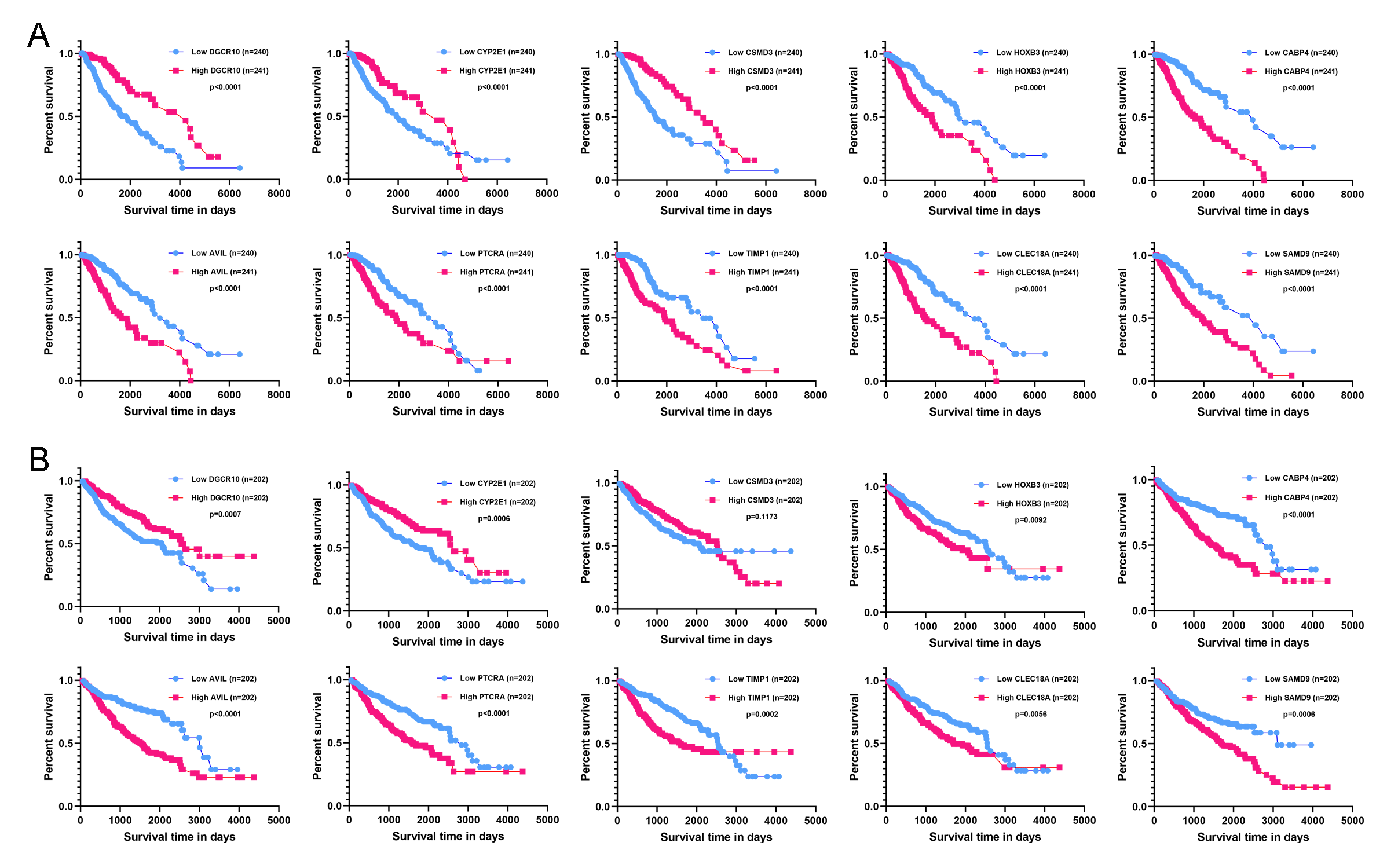

Supplement: Supplementary file 3 — Additional file 3: Figure S3. Kaplan-Meier curves of the 10 prognostic genes in TCGA (A) and CGGA (B) datasets. [file 12859_2023_5328_MOESM3_ESM.tif]

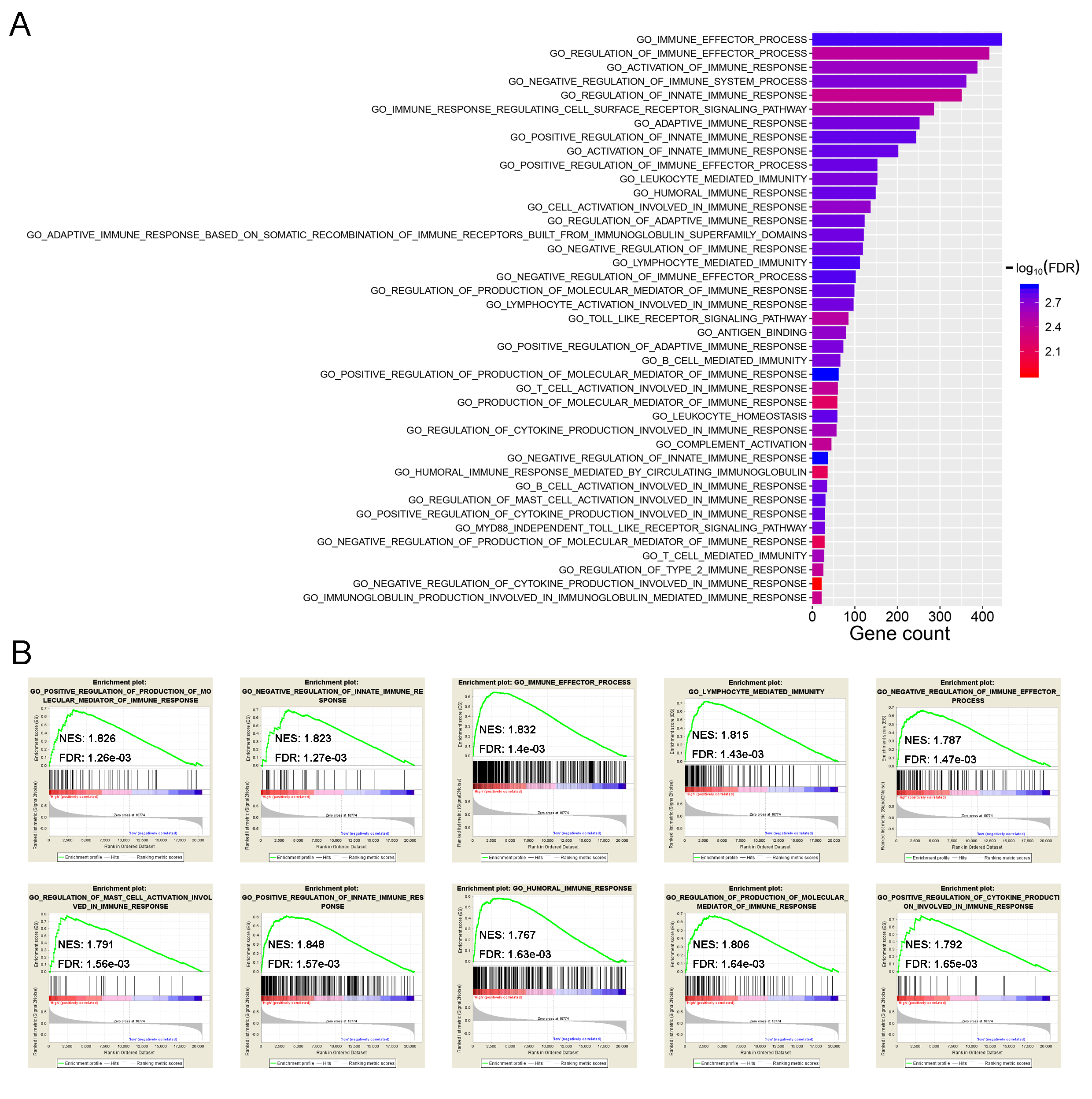

Supplement: Supplementary file 4 — Additional file 4: Figure S4. GSEA showed the immune-related GO terms between low- and high-risk groups. (A) A total of 41 immune-related GO terms were significantly enriched in the high-risk group. (B) The visualization of the top 10 enrichments in the high-risk group. [file 12859_2023_5328_MOESM4_ESM.tif]

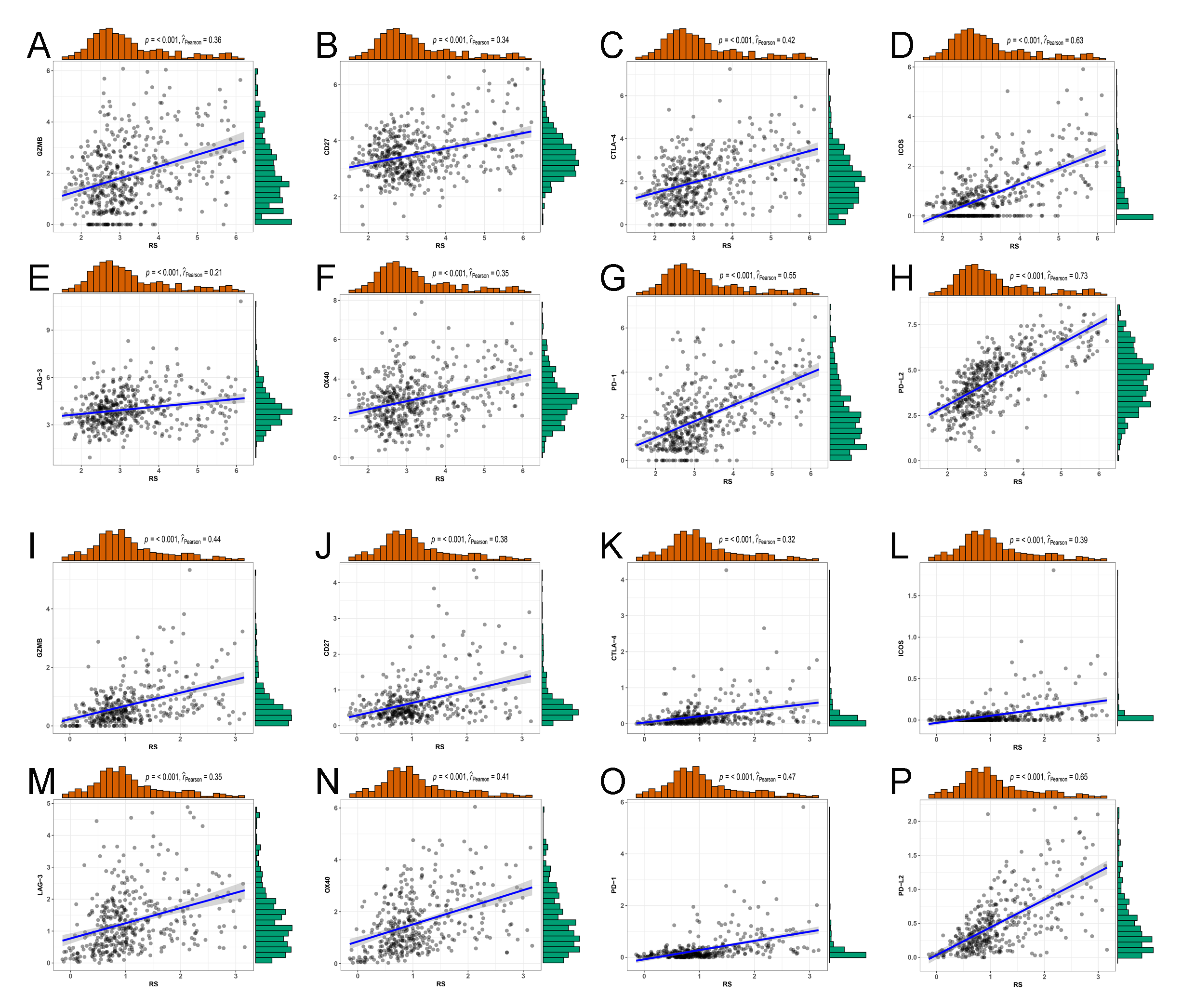

Supplement: Supplementary file 5 — Additional file 5: Figure S5. The correlation analyses between risk score (RS) and immune checkpoints in TCGA (A-H) and CGGA dataset (I-P). [file 12859_2023_5328_MOESM5_ESM.tif]

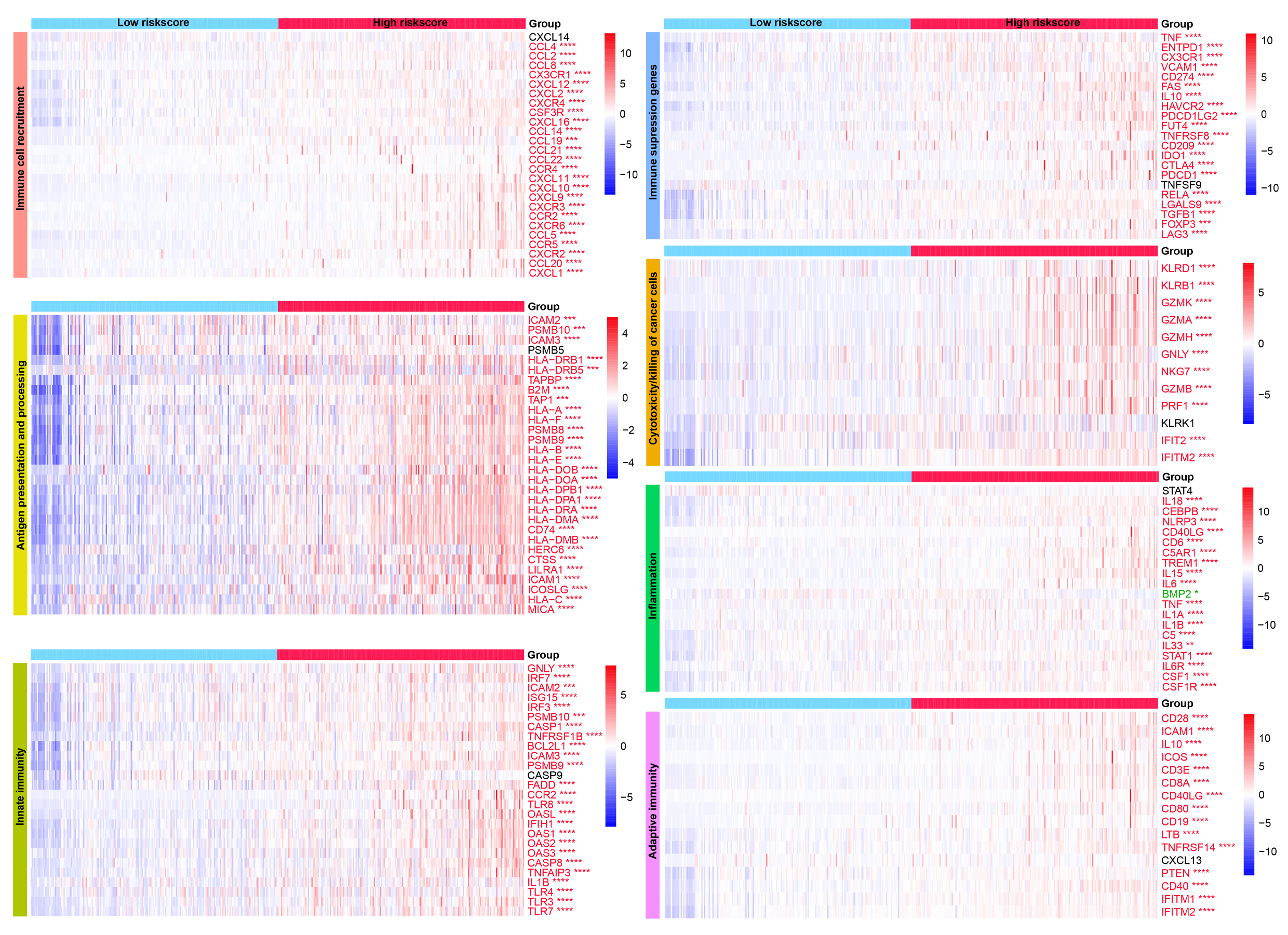

Supplement: Supplementary file 6 — Additional file 6: Figure S6. Activation of several immune pathways in the high-risk groups in the CGGA cohort. These pathways are involved in immune cell recruitment, antigen presentation and processing, innate immunity, immune suppression, cytotoxicity, inflammation, and adaptive immunity. Green font represents the gene overexpressed in the low-risk group, while red represents the gene overexpressed in the high-risk group. Statistical test: Wilcoxon. *, p < 0.05; **, p < 0.01; ***, p < 0.001; ****, p < 0.0001. [file 12859_2023_5328_MOESM6_ESM.tif]

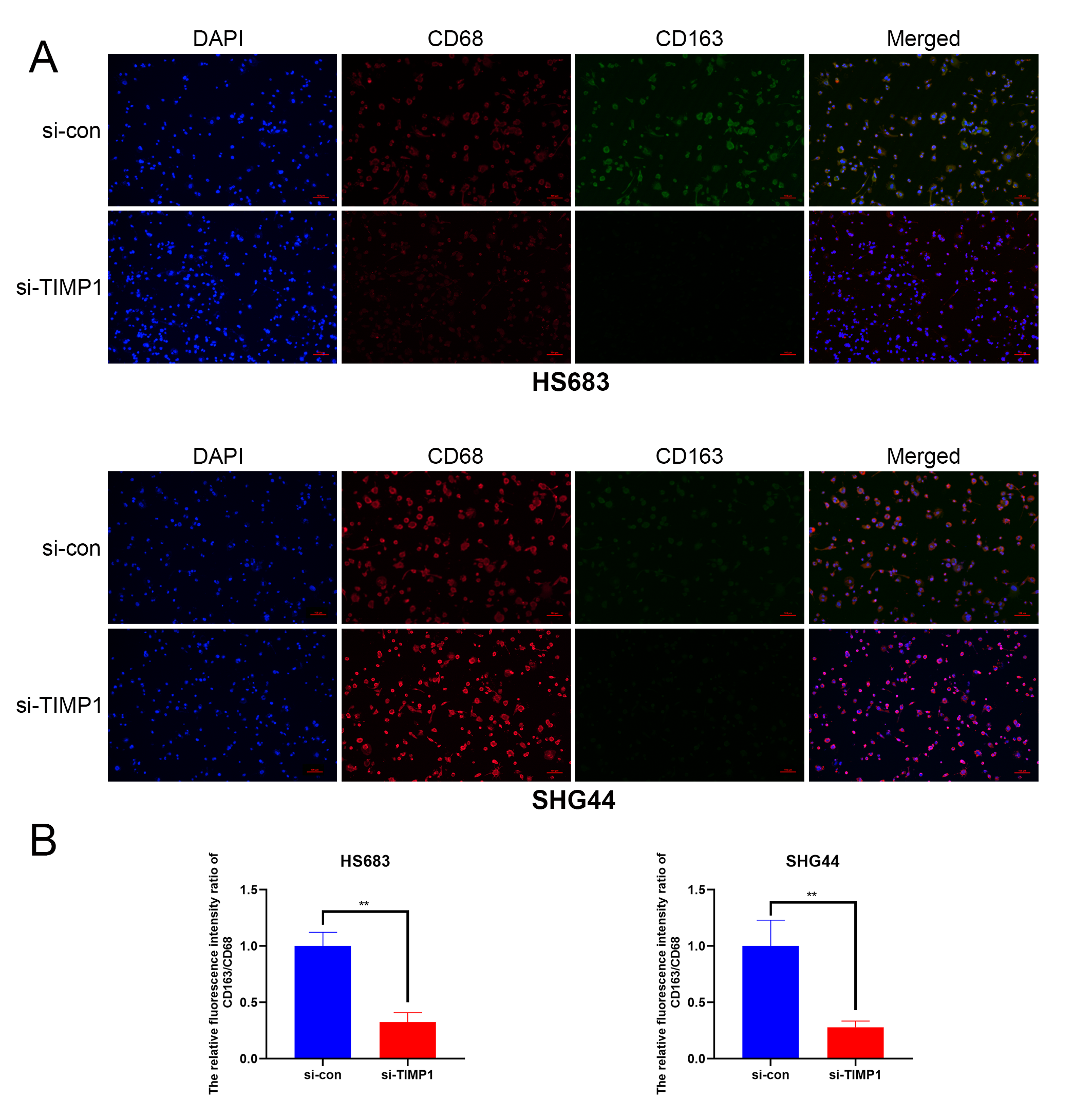

Supplement: Supplementary file 7 — Additional file 7: Figure S7. TIMP1 promotes macrophage differentiation toward M2 in vitro. (A) The expression of CD68 and CD163 in macrophages treated differently detected by immunofluorescence. (B) Statistical analysis of different groups (** Represents p < 0.01). [file 12859_2023_5328_MOESM7_ESM.tif]
